# Using serological measures to estimate influenza incidence in the presence of secular trends in exposure and immuno‐modulation of antibody response

**DOI:** 10.1111/irv.12807

**Published:** 2020-10-27

**Authors:** Talia M. Quandelacy, Derek A. T. Cummings, Chao Qiang Jiang, Bingyi Yang, Kin On Kwok, Byran Dai, Ruiyin Shen, Jonathan M. Read, Huachen Zhu, Yi Guan, Steven Riley, Justin Lessler

**Affiliations:** ^1^ Department of Epidemiology Johns Hopkins Bloomberg School of Public Health Baltimore MD USA; ^2^ Department of Biology University of Florida Gainesville FL USA; ^3^ Guangzhou No. 12 Hospital Guangzhou China; ^4^ The Jockey Club School of Public Health and Primary Care The Chinese University of Hong Kong Hong Kong Special Administrative Region China; ^5^ Stanley Ho Centre for Emerging Infectious Diseases Hong Kong Special Administrative Region The Chinese University of Hong Kong Shatin, Hong Kong China; ^6^ Shenzhen Research Institute The Chinese University of Hong Kong Shenzhen China; ^7^ Center for Health Informatics Computing and Statistics Lancaster Medical School Lancaster University Lancaster UK; ^8^ Institute of Infection and Global Health University of Liverpool Liverpool UK; ^9^ State Key Laboratory of Emerging Infectious Diseases School of Public Health The University of Hong Kong Hong Kong China; ^10^ Shantou University Medical College Shantou China; ^11^ School of Public Health Imperial College London London UK; ^12^ Present address: Centers for Disease Control and Prevention San Juan Puerto Rico

**Keywords:** immunodynamics, incidence, influenza, serology

## Abstract

**Background:**

Influenza infection is often measured by a fourfold antibody titer increase over an influenza season (ie seroconversion). However, this approach may fail when influenza seasons are less distinct as it does not account for transient effects from recent infections.

Here, we present a method to determine seroconversion for non‐paired sera, adjusting for changes in individuals’ antibody titers to influenza due to the transient impact of recent exposures, varied sampling times, and laboratory processes.

**Methods:**

We applied our method using data for five H3N2 strains collected from 942 individuals, aged 2‐90 years, during the first two study visits of the Fluscape cohort study (2009‐2012) in Guangzhou, China.

**Results:**

After adjustment, apparent seroconversion rates for non‐circulating strains decreased while we observed a 20% increase in seroconversion rates to recently circulating strains. When examining seroconversion to the most recently circulating strain (A/Brisbane/20/2007) in our study, participants aged under 18, and over 64 had the highest seroconversion rates compared to other age groups.

**Conclusions:**

Our results highlight the need for improved methods when using antibody titers as an endpoint in settings where there is no clear influenza “off” season. Methods, like those presented here, that use titers from circulating and non‐circulating strains may be key.

## 1 INTRODUCTION

Viral detection is the gold standard for measuring incident influenza infections.^1^, ^2^ Yet, high asymptomatic infection rates and transient viral presence during infections^3^, ^4^ limit detection when using virologic outcomes to measure population‐based influenza burden. Fourfold antibody titer increases over time (ie, seroconversion) and is traditionally used to measure influenza incidence.^1^, ^2^, ^5^ However, an individual's immunological response to influenza combines previous and recent influenza exposures.^6^, ^7^, ^8^, ^9^ Current seroconversion methods often do not account for these effects.

**FIGURE 1:**
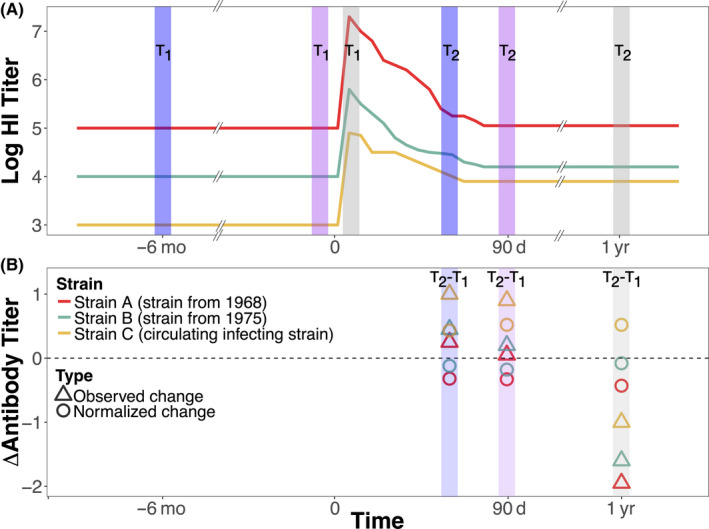
Hypothetical changes in antibody titers to three influenza A H3N2 strains at three sampling sets. A, Lines represent hypothetical changes in log hemagglutination inhibition (HI) antibody titers of three infecting strains, where infections occurred at different points during a lifetime. B, shows antibody changes normalized to Strain A (the oldest hypothetical strain to infect an individual). Vertical lines of the same color represent first and second study visit sera sampling points

Longitudinal sera sampling capture different antibody titer snapshots. Figure 1 illustrates antibody variations before and after infection. Figure 1A shows hypothetical log hemagglutinin inhibition (HI) titers to three influenza strains, sampled antibody response sets over 2 years, and corresponding observed antibody titer changes (Figure 1B). Ideally, initial sampling captures sera before infection (ie, baseline sera) (Figure 1, $T_{1}$ pink vertical lines), and secondary sampling captures post‐infection titers (ie, titers decline to new baselines) (Figure 1, $T_{2}$ pink vertical lines). However, variable sampling times capture different infection points, particularly in communities with weak influenza seasonality. For example, year one sampling captures baseline titers (ie prior to any exposure or infection) (Figure 1, purple $T_{1}$ line), but serological testing captures waning antibodies in year two sampling (Figure 1, purple $T_{2}$ line). In another scenario, year one sampling captures antibodies at their highest concentration to infection (Figure 1, gray $T_{1}$ line) followed by later year two sampling when antibody titers stabilized to new baseline levels (Figure 1, gray $T_{2}$ line). These measured antibody titer differences can inform whether infection occurred between two sampling time points.

Traditional paired sera (ie, sera sampled at two separate points but tested simultaneously) are ideal, but not always logistically feasible for disease surveillance and longitudinal cohort studies. Using independently tested sera can lead to increased assay‐associated error, for example, testing time, protocol adherence, reagent lots, and batch effects, but may be more logistically feasible.^10^ Infection also affects the distribution of titer changes, due to possible influences like prior exposure or temporary boosting. One probabilistic approach accounts for age to estimate infections,^11^ but this approach is computationally intensive and challenging in field settings. Additional adjustment methods are needed for better across‐sample comparisons.

Here, we present an adjustment method using log2 HI titers from multiple recent and historical influenza strains to define seroconversion and measure incident infections. This approach examines the measurement variability in HI serological assays by using the mean titer change. When accounting for an individual's mean titer change, the change to the most recent influenza strain provides a good measure of recent infection while accounting for temporary boosting effects. We apply our method to the FluScape longitudinal study in Guangzhou, China,^12^ separately use adjusted incidence estimates to examine demographic effects on the risk of recent influenza infection, and validate our method by applying simulated titer data using a model by Kucharski et al.^11^

## 2 METHODS

### 2.1 Ethics

Johns Hopkins University, University of Florida, and University of Liverpool Institutional Review Boards approved study protocols and materials. Adults, 18 years and older, provided written consent. Children, 2‐17 years, provided verbal assent, and parents or guardians gave written consent.

### 2.2 Data

Fluscape study participants provided demographic and serological data during the first (December 2009 to January 2011) and second (June 2011 to May 2012) rolling study visits, as previously described.^12^ Briefly, eligible individuals were 2 years and older, residing in selected households from 40 randomly sampled communities around Guangzhou, China. Household, individual, and contact questionnaires, and sera were collected at each study visit. Individual questionnaires collected age, gender, occupational status, health‐related behaviors, influenza vaccination status, and recent influenza‐like illness data.

### 2.3 Laboratory tests

Laboratory methods are previously described.^13^, ^14^ Briefly, HI assays tested non‐paired sera (ie, sera from first and second visits were tested at different times) for antibodies to five historical and recently circulating influenza A H3N2 subtypes (A/Hong Kong/1/1968, A/Bangkok/1/1979, A/Wuhan/359/1995, A/Fujian/411/2002, and A/Brisbane/20/2007), reflecting vaccine strains spread evenly since the A/H3N2/1968 strain emerged.^9^, ^13^, ^15^ More participants’ sera were tested for A/H3N2 compared to A/H1N1 and B during the two visits; therefore, we used H3N2. We measured duplicate antibody titers using twofold serial dilutions from 1:10 to 1:1280. Positive and negative control sera were also tested.

### 2.4 Seroconversion

For each individual, we estimated the change in log2 HI antibody titers between baseline and first follow‐up visits. *Standard* seroconversion was a fourfold increase in HI antibody titers (ie, two‐unit increase in log2 antibody titers). An individual's estimated adjusted log2 titer change (AC) for each strain was defined as the strain‐specific titer change, centered by an individual's mean titer change across all strains:

(1) $${\text{AC}}_{\text{ij}}=\left(T_{i,j,2}‐T_{i,j,1}\right)‐\frac{1}{K}x\sum_{k}\left(T_{i,j,2}‐T_{i,j,1}\right)$$

where the titer (*T*) for i^th^ individuals {i = 1…n} for study visits 1 and 2, j is the influenza A/H3N2 subtype, and K is the number of subtypes tested. We assumed an individual's true baseline titer to older H3N2 strains was less affected by recent infection (ie, is only affected by transient and batch effects), whereas recent infection should increase antibody titers to the most recent strains. We propose that mean‐centering individuals’ strain‐specific antibody titer change across all strains gives a more accurate estimate of changes generated by recent infection. Therefore, mean‐centering an individual's strain‐specific log2 antibody titer change by their antibody titer change to older strains accounts for temporary boosting and artificial changes from serological testing variations (Figure 1). Evaluation of two additional methods found no qualitative differences from the proposed method presented here (Figure S1).

To estimate adjusted seroconversion, we defined a threshold as 2‐standard deviations in log2 titer changes from the oldest strain. A/Hong Kong/1/1968, the reference strain, is the most antigenically distant strain relative to other tested H3N2 strains potentially infecting participants.

### 2.5 Statistical analysis

Chi‐square tests compared baseline characteristics (ie, gender, age, vaccination status, children in household, and residence) by seroconversion. Age group was defined based on influenza risk groups^16^: children (<18 years), adults (18‐49 years), older adults (50‐64 years), and elderly (≥65 years). Vaccination status was defined as ever receiving an influenza vaccine. Households with children were defined as individuals residing in a household with at least one child (<18 years old). For households with only one child, that child's household exposure status was defined as not residing with another because they would not be an exposure risk to themselves.

We compared standard and adjusted methods using strain‐specific antibody titer changes between study visits, and seroconversion rates (SR) and rate ratios (SRR). We examined A/Hong Kong/1/1968 and A/Brisbane/20/2007 titer changes by sampling month to identify trends in titer changes from sampling times. Seroconversion rates (SR) were the proportion of participants seroconverting among participants with available sera for all 5 H3N2 strains at both visits. Seroconversion rate ratios (SRR) compared adjusted and standard seroconversion rates, where 1.0 reflected no methodological difference in estimated seroconversion rates. Exact binomial 95% confidence intervals (CI) were calculated for all rates and rate ratios.^17^ Since infection risk varies by age and children increase risk of influenza transmission,^1^, ^2^, ^18^ we also compared seroconversion rates across age groups and household status. Seroconversions to older strains are believed to result from cross‐reactivity because the only H3N2 strain circulating during the study was the A/Brisbane/2007 strain.

Log‐odds of seroconversion was modeled as a function of age, gender, vaccination, and children in households using logistic regression. We evaluated the association of age to seroconversion using generalized additive models to estimate spline terms (mgcv package).^19^ Age‐specific splines used penalized thin‐plate regression splines, where estimated degrees of freedom (edf) of knots used penalized likelihood maximization. We also examined the interaction of age groups and presence or absence of children in households on seroconversion. Model fits were assessed using Akaike Information Criteria (AIC). Binomial normal approximation and model coefficient standard errors estimated 95% confidence intervals for seroconversion rates and odds ratios (OR).

We tested our statistical adjustment using simulated data and a published model of influenza antibody titers due to multiple sequential exposures.^11^ Six scenarios were simulated using data from Vietnam and China (separate datasets than the one analyzed here). Simulations included the effects of cross‐reactivity from antigenic similarity, long‐ and short‐term antibody boosting generated in previous infections due to subsequent exposure, waning, and antigenic seniority (where previous immunity suppresses responses to subsequent infections). We generated 500 stochastic realizations for 1000 individuals to each scenario and analyzed the data using our adjustment approach described above. Additional simulation details are described in the supplement.

All statistical analyses used R statistical package, version 3.2.4 (https://www.R-project.org).

### 2.6 Sensitivity analysis

We assessed seroconversion rates by vaccination status and seroconversion methods. To identify potential sampling time effects, we examined months between study visits (linear‐term) in age‐adjusted models of recent infection. Effects of gender and vaccination status on recent infection risk were also evaluated.

## 3 RESULTS

### 3.1 Study participant characteristics

During two study visits, 2012 participants from 856 households provided household and individual demographic data. 1018 participants had paired sera available for antibody testing. Previous comparisons found no demographic differences between those who did and did not provide sera.^12^ Of those with paired sera, 942 participants had sera available for all five A/H3N2 strains for both visits (Table 1). Participants were similar in gender, baseline vaccination status, having children in households, and residence location across standard and adjusted strain‐specific seroconversions (Table 1). At baseline, few participants reported ever being vaccinated, and 12% had missing vaccination history (Table 1). Age‐specific seroconversion rates differed by method for the 1968, 2002, and 2007 strains.

**TABLE 1:** Number of A/H3N2 seroconversions (%) among study participants by baseline characteristics

A/H3N2 Strain	2007				2002				1968				Other strains			
Method	Standard	*P*	Adjusted	*P*	Standard	*P*	Adjusted	*P*	Standard	*P*	Adjusted	*P*	Standard	*P*	Adjusted	*P*
No. SCV/Total	264/942		323/942		28/942		8/942		57/942		20/942		86/942		48/942	
Gender																
Male (n = 496)	143 (27%)	.61	167 (34%)	.72	16 (3%)	.77	4 (1%)	>.99	40 (8%)	.01	14 (3%)	.18	50 (4%)	.35	28 (3%)	.51
Female (n = 446)	121 (29%)		156 (35%)		12 (3%)		4 (1%)		17 (4%)		6 (1%)		36 (5%)		20 (2%)	
Age groups (years)																
<18 (n = 73)	27 (37%)	.14	35 (48%)	.01	1 (1%)	.75	2 (3%)	.12	3 (4%)	.25	1 (1%)	.21	7 (5%)	.04	5 (3%)	.09
18‐49 (n = 506)	130 (26%)		147 (29%)		16 (3%)		3 (<1%)		35 (7%)		15 (3%)		58 (6%)		33 (3%)	
50‐64 (n = 263)	74 (28%)		100 (38%)		9 (3%)		1 (<1%)		17 (6%)		4 (2%)		14 (3%)		7 (1%)	
≥65 (n = 100)	33 (33%)		41 (41%)		2 (2%)		2 (2%)		2 (2%)		0 (0%)		7 (4%)		3 (2%)	
Ever received influenza vaccine																
Yes (n = 764)	18 (29%)	.98	22 (35%)	.84	1 (2%)	.95	2 (3%)	.10	5 (8%)	.68	1 (2%)	>.99	5 (4%)	.70	3 (3%)	>.99
No (n = 62)^a^	214 (28%)		255 (33%)		20 (3%)		4 (1%)		45 (6%)		17 (2%)		68 (4%)		39 (2%)	
Households with children																
Yes (n = 202)	66 (33%)	0.12	80 (40%)	.09	7 (3%)	.82	3 (1%)	.50	12 (6%)	.99	6 (3%)	.50	17 (4%)	.80	9 (2%)	.47
No (n = 740)	198 (27%)		243 (33%)		21 (3%)		5 (1%)		45 (6%)		14 (2%)		69 (5%)		39 (3%)	
Location of residence																
Urban (n = 221)	58 (26%)	0.56	87 (39%)	.08	3 (1%)	0.16	3 (1%)	.60	14 (6%)	.97	6 (3%)	.67	30 (7%)	.02	24 (5%)	<.01
Rural (n = 721)	206 (29%)		236 (33%)		25 (3%)		5 (1%)		43 (6%)		14 (2%)		56 (4%)		24 (2%)	

Note

Standard and adjusted seroconversion methods (Equation 1) are those presented in the Methods section. Seroconversion events were combined for the A/Bangkok/1979 and A/Wuhan/1995 strains. *P* refers to chi‐square test for independence *P*‐value, n is the number of individuals in each sub‐group.

Abbreviations: No. SCV, Number of seroconversions.

^a^ 116 individuals did not provide influenza vaccination status at baseline (12% of participants).

### 3.2 Strain‐specific antibody titer changes

**FIGURE 2:**
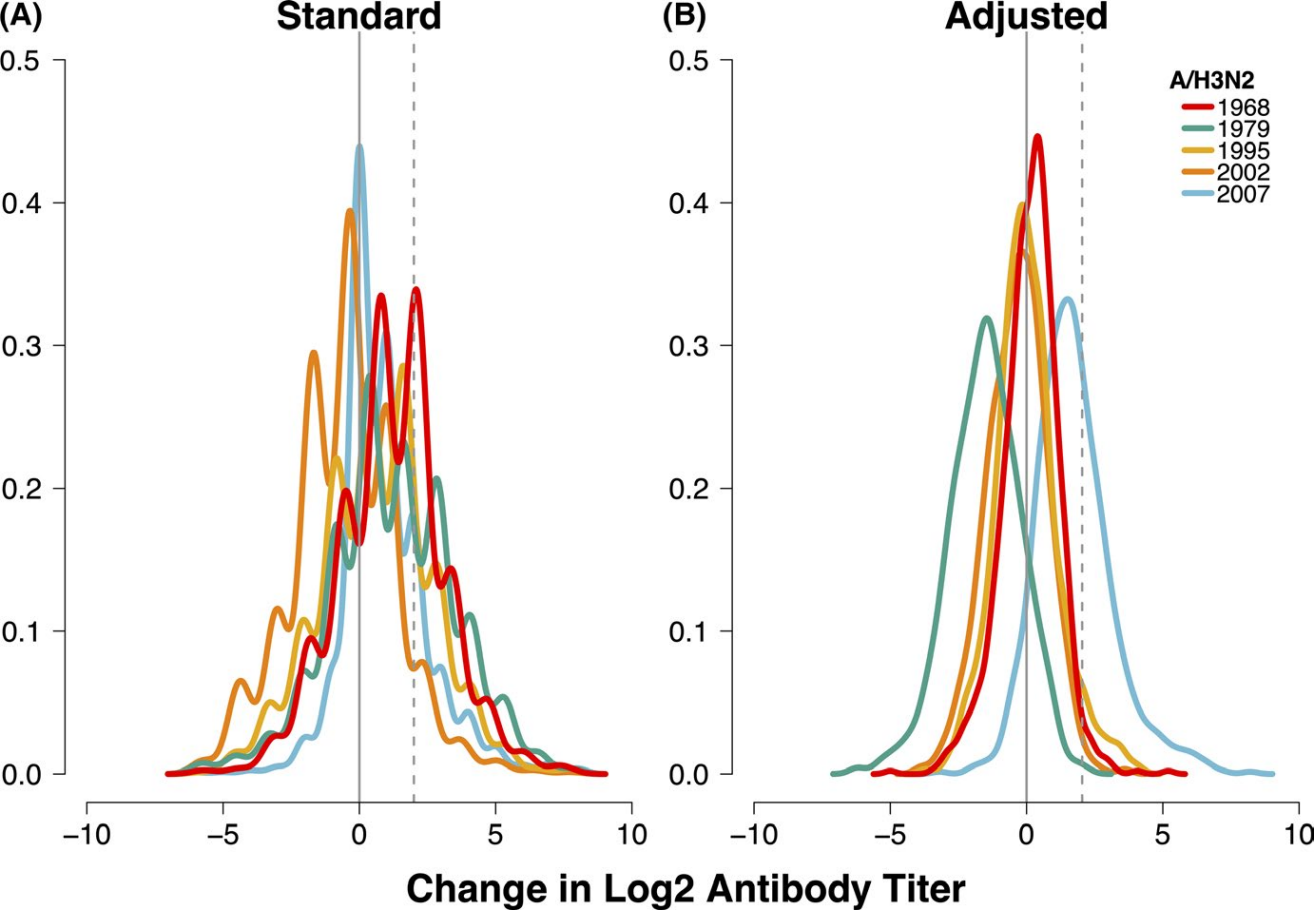
Distribution of H3N2 antibody titer changes from baseline to first follow‐up visit samples from study participants (N = 942). Colors represent five historical and recent influenza A/H3N2 strains. A, shows the distribution of titers changes and (B) shows the distribution of adjusted titer changes. Vertical lines represent no antibody titer changes (black) and seroconversion (SCV) thresholds (dashed)

Overall, when examining mean titer changes across the five strains, the distribution from the standard method was left‐skewed and decreased between visits (mean:‐0.8 (interquartile range (IQR):‐2.0, 0.0)(Figure 2A). A/Bangkok/1/1979 titers decreased the most (mean:‐2.3, (IQR:‐3.0, −1.0)), whereas A/Brisbane/20/2007 titers increased (mean:0.9, (IQR:0.0, 2.0)). After adjustment, most strains’ antibody titers minimally changed between study visits, (Figure 2B), and the initial left‐skewed distribution became more normally distributed (mean:0.0, (IQR:‐1.0, 1.0)). The decreased titers for A/Bangkok/1/1979 remained after adjustment, though at a lower magnitude compared to the standard method. Similarly, the overall change in A/Brisbane/20/2007 antibody titers remained higher after adjustment and increased in magnitude.

**FIGURE 3:**
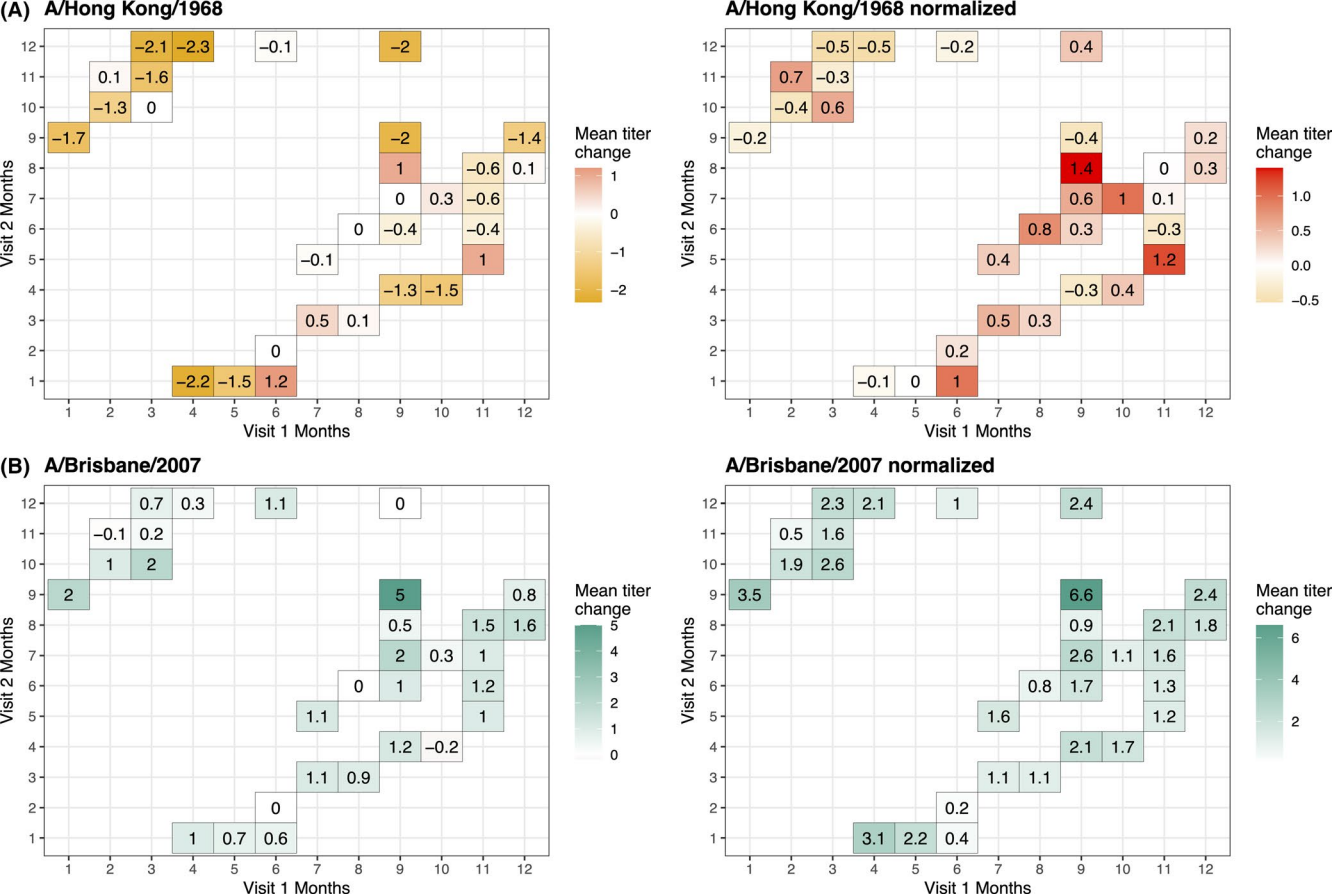
Average monthly titer changes by visit 1 and 2 for standard and adjusted log HI titers. A, Standard and adjusted log HI titers for A/Hong Kong/1968 strain (red). B, Standard and adjusted log HI titers for A/Brisbane/2007 (blue). Gradient colors reflect the magnitude of titer change. Zero reflects no change

Sampling times had no obvious effects on mean titer changes, though titers varied across first and second visits A/Hong Kong/1/1968 and A/Brisbane/20/2007 (Figure 3). The highest mean A/Hong Kong/1/1968 titers occurred among those sampled in January (1st visit) and June (2nd visit) using the standard method, and in August (1st visit) and September (2nd visit) using the adjusted method. In contrast, the highest mean A/Brisbane/20/2007 titer changes occurred in September (1st and 2nd visit) using both methods.

### 3.3 Comparison of standard and adjusted seroconversion rates

The adjusted A/Brisbane/20/2007 strain seroconversion rate, a recent infection proxy, was 1.2 (95% CI:1.1, 1.4) times higher than the standard method, suggesting a 34.3% (95% CI:31.3%, 37.4%) seroconversion for recent infection (Table 2). Overall, 1968‐2002 strains had low seroconversion rates (<10%) using both methods. Lower adjusted seroconversion rates (0.1%‐7.5%) indicate our adjustment removed issues associated with error and accounted for decreasing titers due to relatively recent exposures prior to baseline measurement.

**TABLE 2:** Strain‐specific standard and adjusted seroconversion rates (SR) and ratios (SRR (95% confidence interval (CI))

Method	Standard		Adjusted		Seroconversion rate ratio (SRR) (95% CI)
Strain	Cases	SR (95% CI)	Cases	SR (95% CI)	
A/Brisbane/20/2007	264	28.0% (25.3%, 31.0%)	323	34.3% (31.3%, 37.4%)	1.2 (1.1, 1.4)
A/Fujian/411/2002	28	3.0% (2.1%, 4.3%)	8	0.8% (0.4%, 1.7%)	0.3 (0.1, 0.6)
A/Wuhan/359/1995	71	7.5% (6.0%, 9.4%)	47	5.0% (3.8%, 6.6%)	0.7 (0.5, 0.9)
A/Bangkok/1/1979	15	1.6% (1.0%, 2.6%)	1	0.1% (0.0%, 0.6%)	0.0 (0.0, 0.5)
A/Hong Kong/1/1968	57	6.1% (4.7%, 7.8%)	20	2.1% (1.4%, 3.3%)	0.4 (0.2, 0.6)

Note

Nt: Seroconversion rate ratio compares adjusted method rates to standard method rates. SRR = 1 shows no difference between methods.

Abbreviations: CI, confidence interval; SR, Seroconversion rate; SRR, Seroconversion rate ratio.

Seroconversion rates varied by age groups, and by presence of children in households, though these differences were not significant (Table 3). Adjusted A/Brisbane/20/2007 seroconversion rates were higher by age group compared to standard method rates. Children (<18 years) had the highest seroconversion rates, whereas adults had the lowest rates (standard: 26% (95% CI:22%, 30%) and adjusted: 29% (95% CI:25%, 33%)) (Table 3). In households with children, other children (50%, (95% CI:35%, 65%)), and older adults (45% (95% CI:31%, 60%)) had seroconversion rates higher than other age groups. In households without (other) children present, children (45% (95% CI:30%, 62%)) and the elderly (43% (95% CI:33%, 53%)) (Table 3) had the highest seroconversion rates. Lastly, the effect of second visit vaccination status on A/Brisbane/20/2007 seroconversion comparing standard and adjusted methods showed adjusted seroconversion rates were higher, but did not significantly vary by vaccination status (Table S1).

**TABLE 3:** Standard and adjusted A/Brisbane/20/2007 seroconversion rates (SR) (95% confidence interval) among 924 participants by age groups and presence or absence of children in the household

	Method		Household status	No Children (n = 740)
	Standard (n = 264)	Adjusted (n = 323)	Children present (n = 202)	
Age group (in years)	SR (95% CI)	SR (95% CI)	SR (95% CI)	SR (95% CI)
<18 (n = 73)	37% (27%, 48%)	48% (37%, 59%)	50% (35%, 65%)	45% (30%, 62%)
18‐49 (n = 506)	26% (22%, 30%)	29% (25%, 33%)	35% (27%, 45%)	27% (23%, 32%)
50‐64 (n = 263)	28% (23%, 34%)	38% (32%, 44%)	45% (31%, 60%)	37% (31%, 43%)
≥65 (n = 100)	33% (25%, 43%)	41% (32%, 51%)	22% (6%, 55%)	43% (33%, 53%)

Note

Households status refers to households with or without at least one child (<18 y). Comparisons of age groups by household status show the adjusted seroconversion rates.

Abbreviation: CI, confidence interval; n, total in each age group; No., number; SCV, seroconversion; SR, seroconversion rate.

### 3.4 Effects of individual and household factors on the risk of recent infection

**FIGURE 4:**
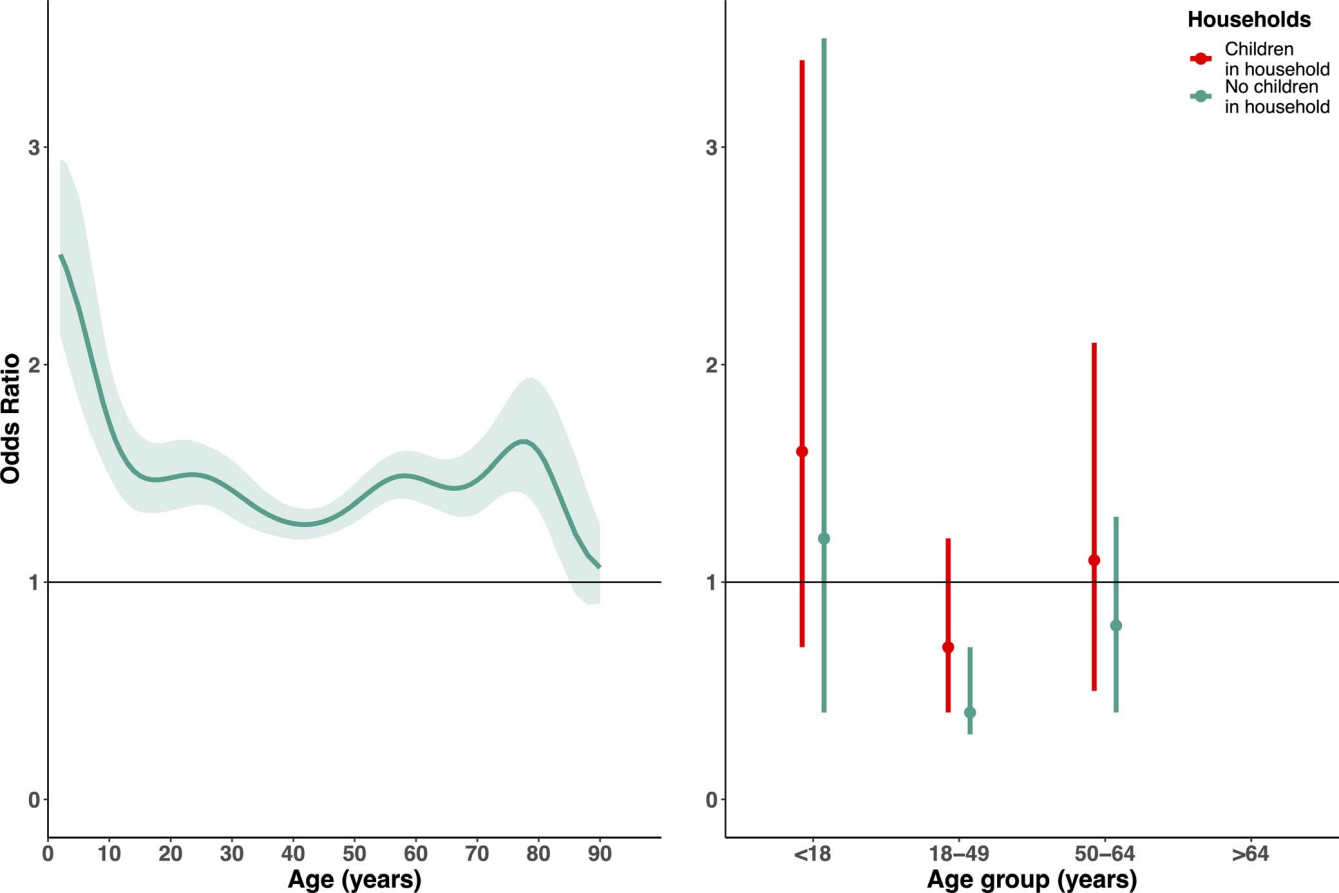
Odds ratios of infection by baseline age, and age groups and household status. A, Odds ratios by baseline age (years) (solid line) and 95% confidence bounds (shading). B, Odds ratios and 95% confidence bounds of recent infection by age group (years) and households with (red) and without (blue) children (<18 y). Due to small numbers, odds ratios were not estimated for 65 and older ages

We independently assessed the risk of infection associated with different household and individual characteristics. Using adjusted A/Brisbane/20/2007 seroconversion as a recent infection proxy, we examined the association of age, sampling month, gender, vaccination status, and presence of children in household with the log‐odds of recent infection. Modeling age non‐linearly (spline edf: 8.1), the risk of recent infection significantly varied by age (*P*‐value: <.01) (Figure 4A). Children and elderly participants had the highest infection risk, while adult risk varied. Time between study sampling showed little effect on risk of recent infection, though model fit improved slightly (Table S2). No association was observed for gender, vaccination status. Individuals in households with children had 48% increased odds of infection compared to those in households without children, adjusting for age (odds ratio:1.48, 95%CI:1.01, 2.14) (Table S3).

Modeling age group and household status interactions showed the risk of recent infection differed (though not significantly except for adults in households without children (*P* < .05)) across age groups based on whether or not they resided in a household with children (likelihood ratio test *P*‐value: .41 comparing the interaction and non‐interaction models of age groups and household terms). The < 18, 18‐49, and 50‐64 year age groups in houses with children had higher odds of infection compared to similar‐aged participants in houses without children (Figure 4B). Among households without children, adults had the lowest risk of infection (odds ratio:0.4 (95% CI:0.3, 0.7), *P*‐value: <.01) and children had the highest risk (odds ratio:1.2 (95% CI:0.4, 3.5). For households with children, odds of recent infection was highest among other children (odds ratio:1.6 (95% CI:0.7, 3.4)) compared to other age groups. We could not evaluate the risk among ≥65 due to small numbers.

### 3.5 Method validation

Our method validation used simulated data from 500 stochastic realizations of 1000 individuals using parameters from Kucharski et al^11^ under six scenarios of differing antigenic seniority and probabilities of infection. Comparing our standard and adjusted seroconversions to the simulated true infection status, standard seroconversion rates were the same and adjusted seroconversion rates were lower than the true infection status with no antigenic seniority present, even at increasing probabilities of infection (Table S6). In the presence of antigenic seniority, both methods underestimated the true infection status, but the adjusted method estimated seroconversion rates 1.5 to 5.6 times higher than standard rates depending on the infection probability, more closely reflecting true infection rates compared to the standard method.

## 4 DISCUSSION

We present a straightforward and transparent statistical adjustment to define recent infection for non‐paired serological data including recent and historical strains. This method identifies recent H3N2 strain seroconversions, accounting for transient immunodynamics and batch effects. This proposed method shows increased A/Brisbane/20/2007 seroconversions, the strain most closely related to those circulating in this population during our study period, and reduced seroconversion rates for older non‐circulating strains, as expected if adjustment was successful. Other methods comparisons like rescaling based on HI assay sensitivity to paired sera^6^, ^20^, ^21^ and simulation approaches to reconstruct paired sera distributions^22^ found standard approaches underestimated seroconversion rates. The risk of A/Brisbane/20/2007 seroconversion was associated with age, and varied by age group and households with children. Our results highlight potential benefits of measuring titers to multiple influenza strains to estimate influenza incidence in cohort studies, particularly in settings with broad seasonality, and suggests using standard seroconversion techniques with single virus strains may underestimate currently circulation strain seroconversion rates.

Children had the highest infection risk, though this estimate had high uncertainty since few children were enrolled. Observed decreasing infections with increasing participant age are similar to findings from a Hong Kong household study during the 2009 pandemic.^18^ Decreased infections among adults (18‐49 years) may be due stronger immune responses to infection, though others have observed higher infection rates among similar age groups.^11^ Older adults (50‐64) and elderly individuals (≥65) had increased infection rates, consistent with increased risk^23^ and associated mortality with increased aged and susceptibility to infection due to co‐morbidities and declining immunity.^24^

Most participants living in households with children had higher associated risks of recent infection. Our finding is consistent with other studies attributing increased influenza transmission to children in households.^1^, ^2^, ^18^ This finding across studies and locations supports an association of increased infection risk with age and households with children,^1^, ^2^, ^18^ even as age groups varied across studies. Adults in households without children had a protective effect, suggesting that less exposure to children, a group with high influenza infections, reduced infection risk. Other studies also found children in households predicted infection or were associated with increased infection risk^18^, ^25^ despite differences in age group definitions (3‐19 years and <9 years),^18^, ^25^ therefore, possibly capturing more infections attributed to younger children than our study (only 6 children under 5 years of age).

Lastly, we found our adjusted seroconversion method performed better than the standard seroconversion method when simulating data where the true infection status was known and antigenic seniority was present. In simulations without impacts of antigenic seniority (see Supplement for details and Kucharski et al^11^), standard methods performed better. However, in simulations with antigenic seniority, the adjusted methods outperformed standard methods. Though adjusted methods still underestimated the true seroconversion rate, adjusted methods were closer to the true seroconversion rates and twice to six times as higher than standard methods.

Our study has limitations. Unable to infer infection using paired sera (ie, sera tested during the same time), we relied on serological titers tested independently (ie, sera from visit 1 and visit 2 tested at different times). However, we proposed a straightforward and easy to interpret method for when paired sera are not available, like in long‐term cohort studies or sero‐surveillance studies. Our method accounted for seroconversions previously misclassified as seronegative based on traditional methods, yet we still observed seroconversions to extinct H3N2 strains. Most study participants were 45 or older, so these individuals possibly had extra boosting still classifying them as seropositive to older non‐circulating strains. Additionally, we used sera tested against five H3N2 strains because these H3N2 strains most frequently circulated, representing the variation of H3 antigenicity since its 1968 emergence.^26^ However, more regionally specific strains may better characterize transient immunodynamics. Additional work is needed to assess how well our method works when applied to other influenza types/subtypes since transient immunodynamics are also associated with H1N1^27^ and influenza B lineages.^28^

Traditional serological studies used a fourfold increase as the standard seroconversion definition. However, this definition can be challenging in longitudinal studies where sampling times vary, and in regions without clearly defined pre‐ and post‐influenza seasons. To our knowledge, other adjustment methods do not fully account for prior influenza exposure, sampling time and laboratory error. Future studies in different settings, using additional historical and recent influenza types/subtypes, are needed to test our method's robustness, given HA group imprinting can provide cross‐immunity.^9^, ^29^ Our methods rely upon having results from multiple strains. We expect our results to work better when more strains are included and expect that our methods may fail if only a small number of strains are used. Accounting for past exposures will be critical in future evaluation of seroconversion, and our method may also apply to other viral families with cross‐reactivity.^30^, ^31^

Our findings show our proposed method for measuring incident influenza infections improves seroconversion estimates to recently circulating influenza strains and in validation, estimated seroconversion rates closer to the true infection rate in the presence of antigenic seniority. Our approach may have relevance for assessing incidence in cohort or sero‐surveillance studies, when testing paired sera maybe logistically challenging. We highlight the need to consider effects of multiple viruses on antibody responses over the life‐course. Since strains circulating earlier in an individual's life continue to influence responses to recent and antigenically‐related subtypes, examining antibody titers to one strain using standard seroconversion methods may fail to fully capture true incident events.

## CONFLICT OF INTEREST

The authors declare that they have no conflicts.

## AUTHOR CONTRIBUTION

**Talia M. Quandelacy:** Conceptualization (lead); Formal analysis (lead); Methodology (equal); Writing‐original draft (lead). **Derek AT Cummings:** Conceptualization (equal); Funding acquisition (lead); Supervision (equal); Writing‐review & editing (equal). **Chao Qiang Jiang:** Funding acquisition (equal); Investigation (equal); Writing‐review & editing (equal). **Bingyi Yang:** Formal analysis (supporting); Writing‐review & editing (supporting). **Kin On Kwok:** Investigation (equal); Writing‐review & editing (supporting). **Byran Dai:** Formal analysis (supporting); Writing‐review & editing (supporting). **Shen Ruiyin:** Investigation (equal); Project administration (equal). **Jonathan M Read:** Funding acquisition (equal); Investigation (equal); Writing‐review & editing (equal). **Huachen Zhu:** Funding acquisition (equal); Investigation (equal); Writing‐review & editing (equal). **Yi Guan:** Funding acquisition (equal); Investigation (equal). **Steven Riley:** Funding acquisition (equal); Investigation (equal); Writing‐review & editing (equal). **Justin Lessler:** Conceptualization (equal); Funding acquisition (equal); Supervision (equal); Writing‐review & editing (equal).

## Supporting information

Supplementary Material
