# Supplementary material for: Using serological measures to estimate influenza incidence in the presence of secular trends in exposure and immuno‐modulation of antibody response
Source: Influenza Other Respir Viruses. 2020 Oct 27;15(2):235–44. doi: 10.1111/irv.12807 (PMC7902255; doi:10.1111/irv.12807)
Supplement: Supplementary file 1 — Supplementary Material [file IRV-15-235-s001.docx]

**Supplemental Information**

We evaluated two additional approaches, in addition to the main adjustment method presented in the Methods section, to evaluate the effect of excluding particular strains from an individual’s mean titer change on their overall individual’s titer change. The two additional approaches are the following:

1) Exclude j^th^ strain from the mean

2) Exclude actively circulating strains from the mean

The first approach estimates an individual’s titer change of j^th^ strain when the mean titer change excludes that j^th^ strain. The second approach estimates an individual’s titer change of j^th^ strain when the recent circulating strain (i.e. A/H3N2/Brisbane/20/2007) is excluded from the mean titer change. We found no qualitative difference when comparing these two approaches to our adjustment method (Supplemental Figure 1A and 1B) and to each other (Supplemental Figure 1C).


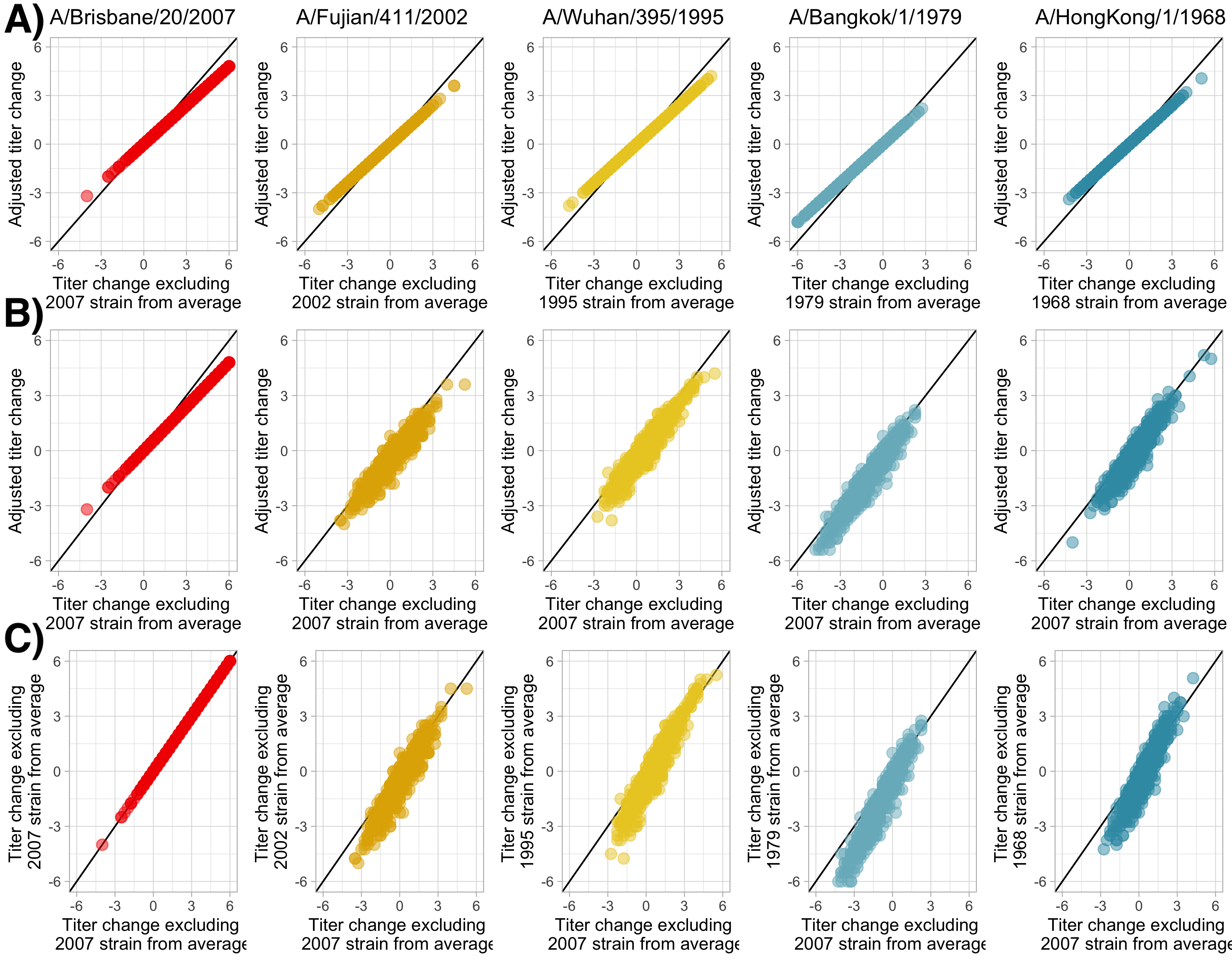


**Supplemental Figure 1. Influenza A/H3N2 strain-specific titer changes comparing three approaches.** A) Adjusted titer changes compared to titer changes when excluding the j^th^ strain of interest from the mean titer change. B) Adjusted titer changes compared to titer changes when excluding the recently circulating strain (A/Brisbane/20/2007) from the mean titer change. C) Titer changes when excluding the j^th^ stain of interest from the mean change compared to titer changes when excluding the recently circulating strain from the mean change.

**Sensitivity Analyses**

We compared how reported vaccination status in the second study changes when comparing standard and adjusted seroconversion estimates. Among those who seroconverted according to the standard method, those who reported never receiving the influenza vaccine as of the second study visit had higher seroconversion rates compared to those who reported ever receiving the vaccine, though these differences were not significant (Supplementary Table 1). While vaccination rates were higher using the adjusted method compared to standard methods, participants who reported ever receiving the vaccine had a slightly higher seroconversion rate compared to those who didn’t receive the vaccine. However, this difference in rates was not statistically significant.

**Supplemental Table 1. Standard and adjusted seroconversion rates (SR) (95% Confidence Interval) to A/Brisbane/2007 by vaccination status during the 2^nd^ study visit**

| **Method** | **Standard** | **Adjusted** |
| --- | --- | --- |
| **Vaccination status*** | **SR (95% CI)** | **SR (95% CI)** |
| Ever received vaccine | 24.6% (15.2%, 37.1%) | 35.1 % (24.0%, 48.1%) |
| Never received vaccine | 28.1% (25.1, 31.1%) | 33.8% (30.7%, 37.1%) |
| Reported seroconversions are to A/Brisbane/2007, a proxy for recent infection. Methods are standard seroconversion methods compared to the adjusted method. Vaccine status reported during the second study visit was defined as ever having received the influenza vaccine or not. Exact binomial confidence intervals are reported. *Forty-six individuals did not report information on vaccination status during the second study visit. Abbreviations: SR, seroconversion rate; CI, confidence interval. | | |

To determine if we adequately adjusted for the sampling month, we examined the effect of including time between study visits (in months) as a potential confounder in our age model for standard and adjusted seroconversion to the 2007 strain. The effect of time in an unadjusted time-only model was not significant in using standard adjusted seroconversion outcome models (Supplemental Table 3), though included in both standard and adjusted seroconversion outcome models adjusting for age, we observed an improved model fit ($\Delta$AIC=-3, and $\Delta$AIC=-8, respectively).

**Supplemental Table 2. Sensitivity analysis of seroconversion to 2007 using standard and adjusted methods examining the effect of time (months) between visits**

| **Method** |  | **Standard** | | **Adjusted** | |
| --- | --- | --- | --- | --- | --- |
| **Model** | **Variables** | **Log odds (β) (95% CI)** | **∆AIC** | **Log odds (β) (95% CI)** | **∆AIC** |
| 1 | Age (ref.) | -- | 0 | -- | 0 |
| 2 | Time (months) between visits | 0.03 (-0.07, 0.14) | 15 | -0.08 (-0.2, 0.03) | 16 |
| 3 | Age | -- | -3 | -- | -8 |
|  | Time (months) between visits | 0.03 (-0.08, 0.13) |  | -0.10 (-0.21, 0.02) | -- |
| Seroconversion models used logistic regression to estimate the log odds of seroconversions. Age was modeled as a non-linear spline term with estimated degrees of freedom using generalized additive models (mgcv package). Months between visits was defined as the number of months between first and second study visits for each participant, and was modeled as a continuous linear variable. The change in AIC uses the age-only model as the reference for each type of seroconversion method, where a reduction in AIC refers to an improved fit compared to the reference (age-only) model. Abbreviations: SCV, seroconversion; CI, confidence interval; β, coefficient; AIC, Akaike’s information criterion; ref., reference. | | | | | |

When examining the effects of gender and baseline vaccination among those who had a reported vaccination status (n=826), we found that neither gender ($\Delta$AIC=2) nor baseline vaccination status ($\Delta$AIC=1) improved fit when included in age models (Supplementary Table 3). Log odds of infection significantly varied by age, and there was no independent effect of households with children on the risk of infection. However, there was an effect of households with children when accounting for the effect of age and in examining model fits, the age-spline and household model had the best fit ($\Delta$AIC: -2) compared to the household-only model ($\Delta$AIC:14), and the age-only model ($\Delta$AIC: 0) (Supplemental Table 3).

**Supplemental Table 3. Effect of gender, household status and vaccination on adjusted H3N2/2007 seroconversion***

| **Model** | **Variables** | **Log odds (β) (95% CI)** | **E.d.f** | **∆AIC** |
| --- | --- | --- | --- | --- |
| 1 | Age (Reference) | -- | 7.9 | 0 |
| 2 | Gender | -0.06 (-0.35, 0.23) |  | 18 |
| 3 | Ever vaccinated | 0.09 (-0.33, 0.51) |  | 18 |
| 4 | Household status | 0.35 (-0.01, 0.70) |  | 14 |
| 5 | Age | -- | 7.9 | 2 |
|  | Gender | -0.10 (-0.39, 0.20) |  |  |
| 6 | Age | -- | 7.8 | 1 |
|  | Ever vaccinated | -0.29 (-0.92, 0.33) |  |  |
| 7 | Age | -- | 8.0 | -2 |
|  | Household status | 0.39 (0.01, 0.76) |  |  |
| Logistic regression models used generalized additive models to estimate the degrees of freedom using thin-plate regression spline terms for age. The reference model used for comparisons of AIC was the age-only model, AIC=1040. Ever vaccinated was defined as ever receiving an influenza vaccine or not at baseline. *There were 826 individuals with a reported baseline vaccination status and therefore model analyses were performed on these individuals. Household status was defined as households with or without at least one child, aged 17 years old or younger. Abbreviations: N, number of observations; CI, confidence interval; β, coefficient; E.d.f, estimated degrees of freedom; ∆AIC, change in Akaike’s Information Criterion. | | | | |

**Simulation methodology**

We generated simulated data to assess the performance of our analysis to quantify seroconversions by using a model of antibody titer that a subset of authors of this paper have published. These models sought to explain present antibody titer status to multiple strains of influenza by modeling a history of past exposure and multiple mechanisms that change titer responses to infecting viruses and other viruses. These mechanisms include: cross-reactivity due to antigenic similarity that operates at short and long time-scales, boosting of titer generated in response to viruses that people have actually been infected by that operate at long and short times-scales, waning of titer and antigenic seniority, a phenomenon that we have hypothesized, where antibody responses to future infections is suppressed by antibody responses to past exposures. Full details of these models are provided in Kucharski *et al.* [12]. Specific values and nomenclature from the Kucharski paper are provided in the Supplemental Table 4 below.

**Supplemental Table 4. Simulation parameters from Kucharski *et al.***

| **Parameter** | **Value** |
| --- | --- |
| Long-term boost (𝜇_1_) | 2.02 |
| Short-term boost (𝜇2) | 2.69 |
| Long-term cross-reaction (𝜎1) | 0.130 |
| Short -term cross-reaction (𝜎2) | 0.031 |
| Waning (𝜔) | 0.79 |
| Antigenic seniority (𝜏) | 0 or 0.04 |

Simulations generated a panel of antibody titers to the Hong Kong 1968 strain, the Bangkok 1979, Wuhan 1995, Fujian 2002 and Brisbane 2007 strains (see main text for full description). For each of six scenarios, we generated 500 stochastic realizations of our simulations for each of 1000 individuals. Each realization provided a titer to each of these five strains at two time points. Simulations also incorporated an infection status for each person and strain.

We performed simulations under the following six scenarios (Supplemental Table 5):

**Supplemental Table 5. Six simulation scenarios**

| **Scenario** | **Antigenic seniority** | **Probability of infection** |
| --- | --- | --- |
| 1 | 𝜏=0 | p=0.2 |
| 2 | 𝜏=0 | p=0.5 |
| 3 | 𝜏=0 | p=0.8 |
| 4 | 𝜏=0.04 | p=0.2 |
| 5 | 𝜏=0.04 | p=0.5 |
| 6 | 𝜏=0.04 | p=0.8 |

We then applied our adjustment method to the simulated data at the two time points to determine whether people seroconverted. We compared this designation by the adjustment method to an unadjusted seroconversion method to the true and know infection status from the simulation. We generated a confusion matrix for each method to quantify the performance.

**Supplemental Table 6. Estimated standard and adjusted seroconversion (SCV) rates using simulated data from 500 stochastic realizations of 1000 individuals based on Kucharski *et al.*** [12]

| **Scenario** | **Antigenic seniority** | **Probability of infection** | **Standard SCV (%)** | **Adjusted SCV (%)** | **True infection status (%)** |
| --- | --- | --- | --- | --- | --- |
| 1 | 𝜏=0 | p=0.2 | 20% | 19% | 20% |
| 2 | 𝜏=0 | p=0.5 | 49% | 25% | 50% |
| 3 | 𝜏=0 | p=0.8 | 80% | 29% | 80% |
| 4 | 𝜏=0.04 | p=0.2 | 13% | 13% | 20% |
| 5 | 𝜏=0.04 | p=0.5 | 9% | 14% | 50% |
| 6 | 𝜏=0.04 | p=0.8 | 3% | 17% | 80% |
